# CRISPRi-Mediated Down-Regulation of the Cinnamate-4-Hydroxylase (C4H) Gene Enhances the Flavonoid Biosynthesis in *Nicotiana tabacum*

**DOI:** 10.3390/biology11081127

**Published:** 2022-07-27

**Authors:** Chou Khai Soong Karlson, Siti Nurfadhlina Mohd Noor, Norzulaani Khalid, Boon Chin Tan

**Affiliations:** 1 Center for Research in Biotechnology for Agriculture (CEBAR), Universiti Malaya, Kuala Lumpur 50603, Malaysia; karlson@um.edu.my (C.K.S.K.); lani@um.edu.my (N.K.); 2 Institute of Microengineering and Nanoelectronics (IMEN), Universiti Kebangsaan Malaysia, Bangi 43600, Malaysia; sitinurfadhlina@ukm.edu.my; 3 Institute of Biological Sciences, Universiti Malaya, Kuala Lumpur 50603, Malaysia

**Keywords:** metabolic engineering, cell suspension culture, CRISPRi, flavonoids, gene silencing

## Abstract

**Simple Summary:**

Flavonoids are natural compounds in plants. They play a critical role in plant growth and pathogen defense. Due to their health benefits, flavonoids have gained much attention as potent therapeutic agents. However, the low abundance of flavonoids in nature has limited their exploitation. Hence, this study aimed to enhance flavonoid production by silencing the cinnamate-4-hydroxylase (C4H) enzyme using the clustered regularly interspaced short palindromic repeats interference (CRISPRi) technology. Our results showed that the *C4H*-silenced tobacco cells had a lower *NtC4H* expression level compared to wild-type. This was concurred by the flavonoid analysis, where the accumulation of C4H’s substrate in the *C4H*-silenced cells was significantly higher than in the wild-type. Our findings provide valuable insight into the future development of CRISPRi to manipulate plant metabolite biosynthesis.

**Abstract:**

Flavonoids are an important class of natural compounds present in plants. However, despite various known biological activities and therapeutic potential, the low abundance of flavonoids in nature limits their development for industrial applications. In this study, we aimed to enhance flavonoid production by silencing cinnamate-4-hydroxylase (C4H), an enzyme involved in the branch point of the flavonoid biosynthetic pathway, using the clustered regularly interspaced short palindromic repeats interference (CRISPRi) approach. We designed three sgRNAs targeting the promoter region of *NtC4H* and cloned them into a CRISPRi construct. After being introduced into *Nicotiana tabacum* cell suspension culture, the transformed cells were sampled for qPCR and liquid chromatography-mass spectrometry analyses. Sixteen of 21 cell lines showed PCR-positive, confirming the presence of the CRISPRi transgene. The *NtC4H* transcript in the transgenic cells was 0.44-fold lower than in the wild-type. In contrast, the flavonoid-related genes in the other branching pathways, such as *Nt4CL* and *NtCHS*, in the *C4H*-silenced cells showed higher expression than wild-type. The upregulation of these genes increased their respective products, including pinostrobin, naringenin, and chlorogenic acid. This study provides valuable insight into the future development of CRISPRi-based metabolic engineering to suppress target genes in plants.

## 1. Introduction

Flavonoids constitute one of the largest groups of secondary metabolites in plants, encompassing more than 6000 identified compounds [1,2]. Their chemical structure consists of a C6-C3-C6 carbon framework, formed by two aromatic rings and a heterocyclic ring containing one oxygen atom [3]. Flavonoids can be broadly categorized into the following six major subgroups: anthocyanins, chalcones, flavones, flavonols, flavandiols, and proanthocyanidins [4]. Many flavonoids have been shown to play important roles in plants. These include giving color and fragrance to flowers [5], attracting pollinators in fruit for seed dispersal [6], growth and development in seedlings [7], signaling during nodulation and auxin transport [8], as well as protecting plants against abiotic stress (e.g., UV radiation, drought, and salt stress) and biotic stress (e.g., insects and herbivores) [9,10]. The protective effects of plant-derived flavonoids against stress are achieved via the secretion or production of specific flavonoids that act as insect/herbivore repellents, phytoalexins, and reactive oxygen species scavengers [11]. 

Flavonoids have been shown to exhibit various therapeutic properties, including antibacterial [12], anti-herpes [13], antioxidant [14], anti-inflammatory [15], anti-ulcer [16], anti-cancer [17], anti-aging [18], and anti-diabetic [19]. Besides, flavonoids (panduratin A and 4-hydroxypanduratin A) extracted from *Boesenbergia rotunda* (medicinal ginger) possess inhibitory activity against Dengue NS2b/3 protease of the dengue virus serotype 2 (DEN-2) [5,20]. This discovery could lead to the potential development of antiviral drugs against dengue since an efficacious antiviral drug has yet to be developed. 

**Figure 1:**
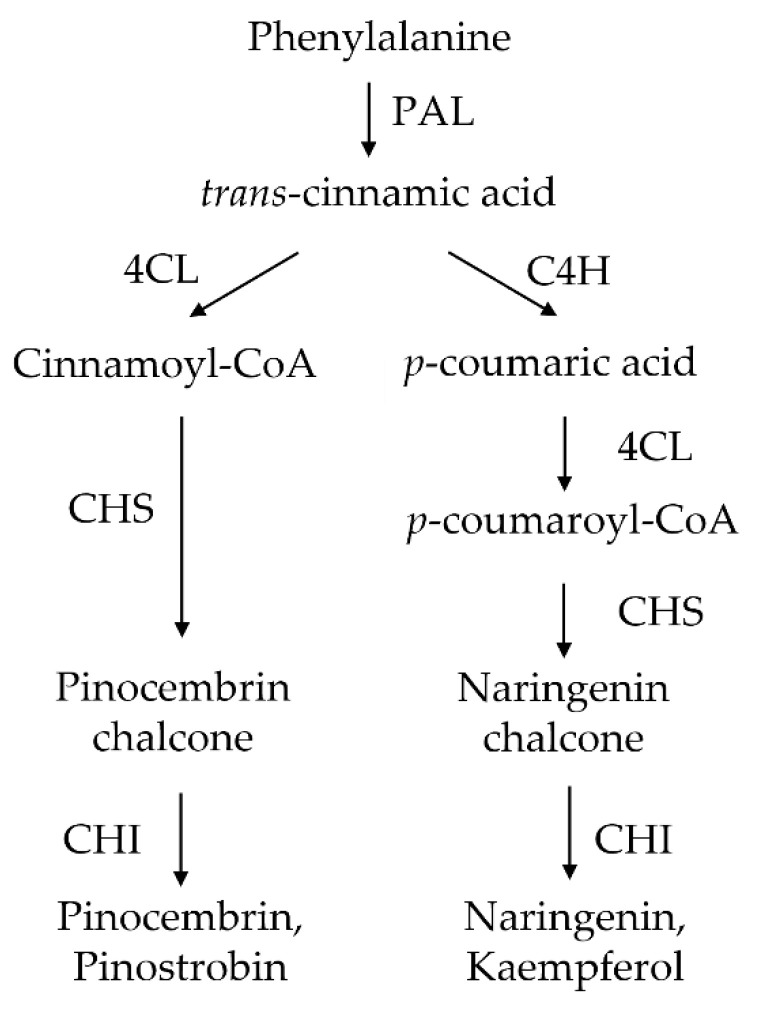
Phenylpropanoid biosynthetic pathway in plants.

In plants, flavonoids are biosynthesized via the general phenylpropanoid pathway (Figure 1), where the processes are tightly regulated by enzymatic reactions [21]. Phenylalanine (synthesized from the shikimate pathway) is the first precursor in the phenylpropanoid pathway. It can be converted to *trans*-cinnamic acid through a deamination reaction by phenylalanine ammonia-lyase (PAL). Later, *trans*-cinnamic acid could be converted to *p*-coumaric acid and cinnamoyl CoA by cinnamate-4-hydroxylase (C4H) and 4-coumarate-CoA ligase (4CL), respectively (Figure 1**)**. Next, chalcone synthase (CHS) catalyzes *p*-coumaroyl CoA or cinnamoyl CoA by condensing malonyl CoA (derived from acetyl-CoA catalyzed by acetyl-CoA carboxylase) at a ratio of 1:3 to form the chalcone precursor. Chalcone isomerase (CHI) then catalyzes the isomerization of chalcone to flavanones, which can be further modified by several enzymes, such as hydroxylases and methyltransferases, to generate various classes of flavonoids. 

Flavonoids are naturally produced in low quantities and are insufficient to meet industrial demands. Furthermore, flavonoid production in plants could be influenced by various factors, such as soil type [22] and climate conditions [23], hindering their potential as valuable candidates for therapeutic applications. However, with a better understanding of the flavonoid biosynthetic pathway and the development of genome editing tools, it has become possible to increase flavonoid production through metabolic engineering approaches. Metabolic engineering approaches can be used to (i) increase the activity of rate-limiting enzyme(s) in upstream pathways, (ii) overexpress the first committed enzyme in the pathway, (iii) block the competing pathways downstream of branch points, and (iv) create sink compartments [24]. For instance, increasing the activity of the rate-limiting enzyme (such as CHS) enhanced the flavonoid production in *Glycyrrhiza uralensis* [25]. Similarly, overexpressing the flavonoid 3′-hydroxylase (F3′H) in poplar increased the accumulations of epigallocatechin, gallocatechin, and catechin [26]. 

In this study, we aimed to enhance flavonoid production by silencing C4H, a cytochrome P450-dependent hydroxylase enzyme (CYP73). We hypothesized that silencing the *C4H* gene in the branching pathway could shift the metabolic flux to the 4CL route. To test our hypothesis, we used the clustered regularly interspaced short palindromic repeats interference (CRISPRi) to silence *NtC4H* in *Nicotiana tabacum* (tobacco) and determine its flavonoid production. The CRISPRi silencing system exploits the deactivated variants of the Cas9 enzyme (dCas9), guided by a sgRNA, to form a dCas9/sgRNA complex that is incapable of cleaving DNA but retains its ability to specifically bind to the DNA [27]. This technology has recently gained immediate attention due to the sequence-specific transcriptional regulation it can offer. In this work, we designed three sgRNAs to target the promoter region of *NtC4H*. These sgRNAs were assembled into a CRISPRi vector and introduced into *N. tabacum* suspension cells via *Agrobacterium tumefaciens*-mediated transformation. The flavonoids in the *C4H*-silenced tobacco cells were harvested for liquid chromatography-mass spectrometry (LC-MS) analysis. 

## 2. Materials and Methods

### 2.1. Plant Material and Establishment of Cell Suspension Culture

In vitro *N. tabacum* L. plantlets obtained from the Plant Biotechnology Research Laboratory, Universiti Malaya were used as explants for callus induction. Under sterile conditions, leaves from *N. tabacum* were cut into 0.5 cm × 0.5 cm segments and cultured on the callus induction medium containing Murashige and Skoog (MS) basal medium [28], 0.1 mg/L α-naphthaleneacetic acid (NAA), 0.1 mg/L benzylaminopurine (BAP), 3% (*w*/*v*) sucrose, and 0.2% (*w*/*v*) Gelrite^®^. The cultures were incubated at 26 ± 2 °C in darkness until friable whitish calli appeared on the leaf explants. To establish cell suspension cultures, the generated *N. tabacum* calli (~ 0.5 g) were transferred to a 250 mL Erlenmeyer flask containing 50 mL TLM [MS basal medium supplemented with 1 mg/L 2,4- dichlorophenoxyacetic acid (2,4-D), 0.04 mg/L kinetin, and 3% (*w*/*v*) sucrose] for 21 days. The pH of all media was adjusted to 5.8 before autoclaving at 121 °C for 20 min. The cell suspension cultures were agitated at 80 rpm on a rotary shaker and sub-cultured every 14 days by replacing the old medium with a fresh liquid medium at a ratio of 1:4. All cultures were incubated at 26 °C in a growth room under a 16-h light and 8-h dark cycle with a light intensity of 31.4 μmol m^−2^ s^−1^ provided by cool fluorescent lights.

### 2.2. sgRNA Design

The promoter region of the *NtC4H* gene in *N. tabacum* was located using the plant promoter prediction program (TSSP) [29]. A 25 nucleotide-specific DNA binding region together with protospacer adjacent motif (PAM) sequence (NGG) at the predicted promoter region were designed for both the template and non-template strands of *NtC4H* using the sgRNA designation program (CRISPOR) [30]. Three sgRNAs with the highest predicted efficiency score and the lowest off-target mismatch were selected and flanked with *EPS3I* (Thermo Scientific, Waltham, MA, USA) restriction site.

### 2.3. Cloning of sgRNAs into Expression Vectors

**Figure 2:**
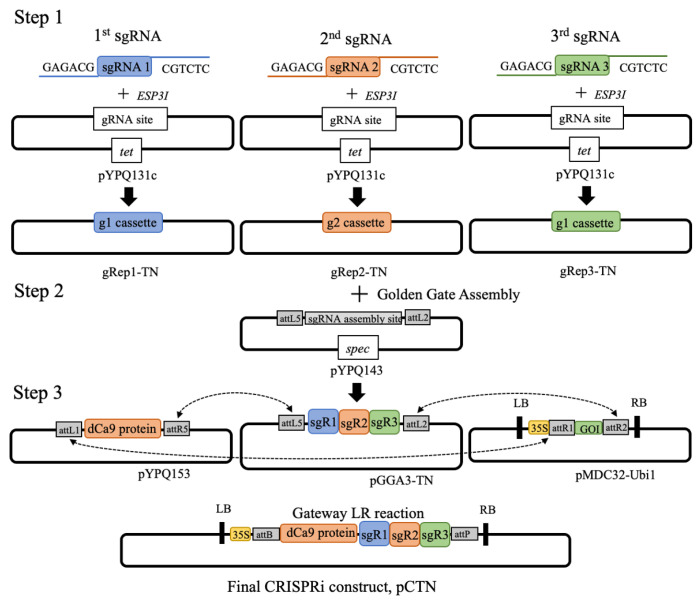
Illustration of the three-step cloning procedure for CRISPRi vector, pCTN, construction using Golden Gate assembly and Gateway LR reaction. Step 1 shows the cloning of individual gRNAs into a series of gRNA expression vectors (pYPQ131c, pYPQ132c, and pYPQ133c) that contain their own promoter. Step 2 involves the assembly of multiple gRNA expression vectors into one Golden Gate recipient vector, pYPQ143 (for three gRNA expression cassettes). Step 3 involves the final assembly of dCas9 vector and gRNA cassette into a single T-DNA binary vector (pMDC32-Ubi1) through Gateway recombination. LB, left border; RB, right border; *spec*, spectinomycin resistance marker; *tet*, tetracycline resistance marker; *kan*, kanamycin resistance marker; GOI, gene of insert.

The construction of the CRISPRi vector containing the three sgRNAs involved a three-step cloning method, illustrated in Figure 2. The first step involved the cloning of the tobacco sgRNA expression vectors (pYPQ131c, pYPQ132c, and pYPQ133c (Addgene, Watertown, MA, USA; Table 1) that contained the sgRNAs as inserts. Prior to transformation, sgRNA expression vectors were first digested with *BglII* and *SalI* followed by *EPS3I* to obtain linearized sgRNA expression vectors. Annealing of the previously designed forward and reverse sgRNA oligos (100 μM) was performed using the T4 Polynucleotide Kinase (NEB, Ipswich, MA, USA). Subsequently, the annealed sgRNAs were diluted with double-distilled water (ddH_2_O) at a ratio of 1:200. sgRNA expression vectors and sgRNAs (inserts) were ligated by T4 DNA ligase (NEB) at room temperature for 1 h to generate three expression vectors, gRep1-TN, gRep2-TN, and gRep3-TN; Table 2). Expression vectors were heat-shock transformed into *Escherichia coli* DH5α [31]. The plasmids from positive colonies were isolated and sequenced using the following primer-specific sequences: 5′-CAA GCC TGA TTG GGA GAA AA-3′ and 5′-GCC AGT GTG ATG GAT ATC TG-3′ to confirm the sgRNA sequence in their respective expression vectors. 

### 2.4. Golden Gate Assembly of Multiple sgRNAs

The second cloning procedure used the Golden Gate Assembly Kit (NEB) to assemble all three sgRNAs into the sgRNA entry vector (Figure 2). The Golden Gate reaction was set up in a 10 μL reaction volume containing 100 ng of each gRNA expression vector (gRep1-TN, gRep2-TN, and gRep3-TN), 1 μL T4 DNA ligase buffer, 100 ng of Golden Gate recipient vector (pYPQ143; Addgene, Watertown, MA, USA; Table 1), 0.5 μL *BsaI* (NEB), and 0.5 μL T4 DNA ligase. The Golden Gate reaction was performed using the following cycling parameters: 10 cycles; 37 °C for 5 min, 16 °C for 10 min, 50 °C for 5 min, and 80 °C for 5 min to produce the sgRNA entry vector (pGGA3-TN; Table 1). After transforming into *E. coli*, the plasmids from positive colonies were isolated and sequenced using the following primer-specific sequences: 5′-CGG CCA GTC TTA AGC TC-3′ and 5′-TAT CAG CTG GAT GGC AAA T-3′ to confirm the presence of all three sgRNAs in the sgRNA entry vectors (pGGA3-TN). 

### 2.5. CRISPRi Vector Construction

The final cloning step involved an LR recombination reaction where the validated sgRNA entry vector (pGGA3-TN) and the dCas9 entry vector (pYPQ153; Addgene, Watertown, MA, USA) were cloned into the plant expression vector (pMDC32-Ubi1; Addgene, Watertown, MA, USA), generating the final CRISPRi vector (pCTN) (Figure 2, Table 1). The reaction was catalyzed by the LR Clonase enzyme mix (Invitrogen, Watertown, MA, USA). The plasmids from positive colonies were sequenced using the following primer-specific sequences: 5′-AGA CTA GTA AGG GCA AAT TC-3′ and 5′-GCG GAT AAC AAT TTC ACA CAG G-3′ and transformed into *A. tumefaciens* strain LBA4404.

### 2.6. Plant Transformation

*A. tumefaciens* harboring pCTN containing *nptII* and *hpt* genes was used to infect *N. tabacum* cell suspension cultures, according to Shumin et al. [32]. The 4-day-old *N. tabacum* cell suspension culture (5 mL) was mixed with the *A. tumefaciens* culture (100 μL) and co-cultivated at 28 °C in darkness for 3 days without shaking. After incubation, the cells were washed twice with 15 mL TLM supplemented with 300 mg/L cefotaxime (Duchefa, Haarlem, The Netherlands) and 100 mg/L carbenicillin (Duchefa, Haarlem, The Netherlands). The *N. tabacum* cells were then resuspended and plated on selection agar TSM (TLM supplemented with 300 mg/L cefotaxime, 100 mg/L carbenicillin, 30 mg/L hygromycin B, 2% (*w*/*v*) Gelrite^®^). After 5–6 weeks of culture, the generated calli were transferred to TSM for propagation. A single colony of callus was selected to be propagated on liquid TSM to obtain a single cell line. The transformation efficiency was calculated and expressed as the number of positive regenerants per total number of independent transformation events.

### 2.7. PCR Amplification

Total genomic DNA from putatively transformed cells was isolated according to the modified procedures by Doyle and Doyle [33]. For PCR analysis, 100 ng total DNA was added to a 20 μL PCR containing 10 μL GoTaq^®^ Master Mixes (Promega, Madison, WI, USA) and 0.1 μM of each primer pair: 5′-AGA CTA GTA AGG GCA AAT TC-3′ and 5′-GCG GAT AAC AAT TTC ACA CAG G-3′. PCR amplification was performed in a thermal cycler with an initial denaturation at 95 °C for 1 min, followed by 30 cycles at 95 °C for 1 min, 50 °C for 1 min, 72 °C for 1 min, and a final extension of 72 °C for 10 min. PCR products were separated on 1.0% agarose gel using electrophoresis and analyzed using a Gel-Pro imager and analyzer (Micro Lambda, Fremont, CA, USA). The product of the pCTN fragment (~405 bp) was confirmed by sequencing.

### 2.8. Quantitative Real-Time PCR (qPCR)

Total RNA was isolated from *N. tabacum* cells based on a modified protocol described by Toni et al. [34]. cDNA was synthesized from 100 ng total RNA using ReverTra Ace qPCR RT master mix (TOYOBO, Osaka, Japan) according to the manufacturer’s instructions. qPCR was carried out using SensiFast SYBR green master mix (Bioline, London, UK) and normalized to the endogenous *EF1α* and *LF25* gene expression. The qPCR primer pairs were designed with PrimerQuest Tool (Integrated DNA Technologies, Coralville, IA, USA), targeting *NtC4H* (Accession No: MW260510), *Nt4CL* (Accession No: D43773), *NtCHS* (Accession No: KF927021), and *NtCHI* (Accession No: KJ730247) genes (Table 3). qPCR conditions were optimized for primer specificity, annealing temperature, and concentration. Each sample was tested in triplicate, and reaction mixtures were prepared according to the manufacturer’s instructions. qPCR assays were performed using the Applied Biosystems 7500 Real-Time PCR machine (ABI, Los Angeles, CA, USA) with the following cycling conditions: 50 °C for 2 min, 95 °C for 20 s, and run at 95 °C for 5 s, and 60 °C for 20 s for 40 cycles. The relative quantification of the gene expression level was calculated using ABI 7500 System Sequence Detection software v1.2 (ABI, Los Angeles, CA, USA).

### 2.9. Extraction of Metabolites 

The transformed *N. tabacum* cells were freeze-dried for about 2 days. About 500 mg of cells were extracted using 5 mL methanol. The mixture was sonicated for 5 min at 37 kHz before incubating at 4 °C overnight. The extraction process was repeated twice. The methanol extracts were evaporated and partitioned with an equal volume of ethyl acetate (EA) and water. The EA fractions were evaporated to dryness, and the crude extracts were dissolved in methanol. All samples were filtered through a 0.45 mm PTFE filter before being analyzed by LC-MS. 

### 2.10. LC-MS Analysis 

The methanol extracts from putative transformed and untransformed cells were analyzed using the Agilent A6490 LC-MS system coupled with a triple quadrupole mass detector. The separation was carried out using a reversed-phase Eclipse plus C 18 column (1.8 μm, 2.1 × 50 mm) with corresponding solvent A [0.1% formic acid (FA; Merck, Kenilworth, NJ, USA) in water] and solvent B [0.1% FA in acetonitrile (Merck, Kenilworth, NJ, USA)]. The solvent gradient started at 95% of solvent A for 11 min and decreased to 40% for 2 min. At the 13th min, the solvent gradient was reduced to 5% A for 6 min before increasing to 95% on the 19th min. The reaction was run for 20 min at a flow rate of 0.2 mL per min. Mass spectra were acquired in multiple reactions monitoring with positive electrospray ionization mode. The concentration of analytes was determined by interpolating the relative peak areas for each analyte to the internal standard peak area in the sample on the spiked calibration curve. An internal standard, collidine, was used to compensate for losses during sample processing and instrumental analysis. The content of flavonoids in each sample was quantified in ng/mL.

### 2.11. Statistical Analysis

Data from qPCR and LC-MS analyses were presented as mean ± standard deviation of three independent experiments. Data analysis of the flavonoid-related gene expression and the differences in compound accumulation in wild-type and *C4H-*silenced cells were compared using paired *t*-tests. A *p*-value of less than 0.05 was considered statistically significant.

## 3. Results

### 3.1. Introducing the CRISPRi Silencing Vector into N. tabacum

**Figure 3:**
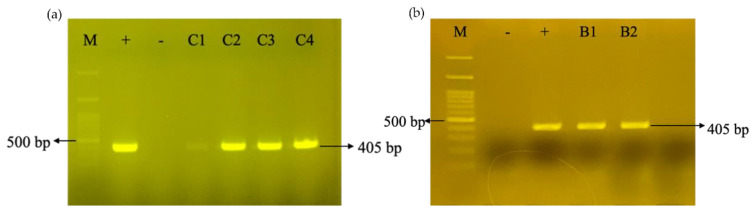
Representative gel electrophoresis image of PCR verification on the short fragment between gRNA entry vector (pGGA3) and the plant expression vector (pUMDC32-Ubi1) in (**a**) CRISPRi vector, pCTN, and (**b**) *Agrobacterium tumefaciens* strain LBA4404 harbouring pCTN. Lane M, 100 bp DNA ladder H3 RTU (GeneDireX, Taiwan, China); Lane -, PCR negative control using double-distilled water as DNA template; Lane +, PCR positive control using plasmid pCTN; Lanes C1–C4, *Escherichia coli* transformed with pCTN; Lanes B1 and B2, *A. tumafaciens* transformed with plasmid pCTN.

The sgRNAs in the gRep1-TN, gRep2-TN, and gRep3-TN vectors were assembled into the sgRNA entry vector (pYPQ143), generating a gRNA entry vector (pGGA3-TN) (Supplementary Figure S1a). The positive clones were confirmed by sequencing (Supplementary Figure S1b). Both the pGGA3-TN and pYPQ153 vectors were subsequently cloned into pUMDC32-Ubi1, producing the final CRISPRi expression vector, pCTN. PCR amplification and sequencing confirmed the presence of a 405 bp fragment that contained the three sgRNAs in the pUMDC32-Ubi1 vector (Figure 3a; Supplementary Figure S2). Finally, the pCTN plant expression vector was transformed into an *N. tabacum* cell suspension culture, in which 16 out of 21 showed an expected amplicon size of 405 bp (Figure 3b). 

### 3.2. Gene Expression of the C4H-Silenced Cells

**Figure 4:**
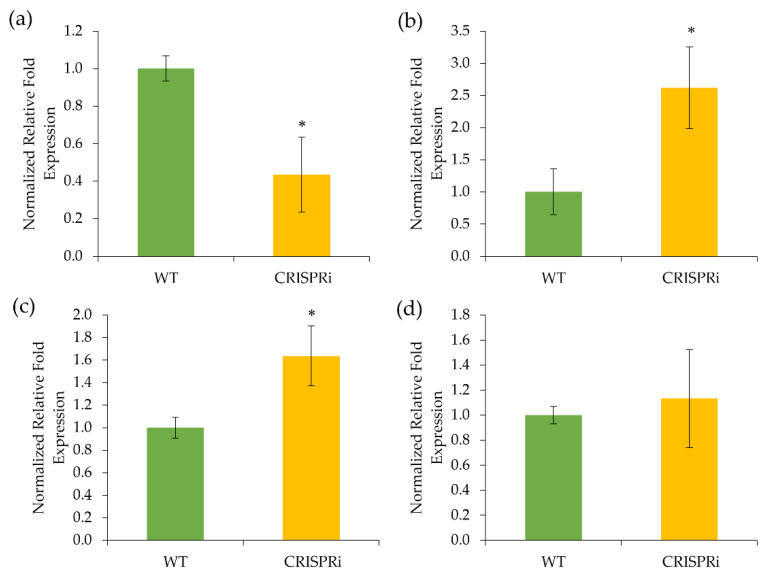
Quantitative real-time PCR analysis of flavonoid-related key enzymes in wild type (WT) and transgenic CRISPRi cell lines of *Nicotiana tabacum*. (**a**) *Cinnamate-4-hydroxylase*, (**b**) *4-coumarate ligase*, (**c**) *chalcone synthase*, and (**d**) *chalcone isomerase*. Data represent the means of three biological replicates (n = 3). Error bars are standard deviation (±SD). Asterisk (*) represents significant differences at *p* < 0.05 according to paired *t*-test.

The *C4H* transcript level for the transformed cell lines was significantly reduced by 0.44-fold compared to wild-type lines (Figure 4a). In addition, after silencing *NtC4H*, the downstream flavonoid-related gene expression of *Nt4CL, NtCHS*, and *NtCHI* was altered. In *C4H*-silenced cells, the expression level of *Nt4CL* and *NtCHS* was 2.62- and 1.64-fold, respectively, higher than in the wild-type cell line (Figure 4b,c). However, there was no statistically significant difference in *NtCHI* expression between the wild-type and the *C4H-*silenced cells (Figure 4d). 

### 3.3. LC-MS Analysis of Flavonoids in the C4H-Silenced Cells 

**Figure 5:**
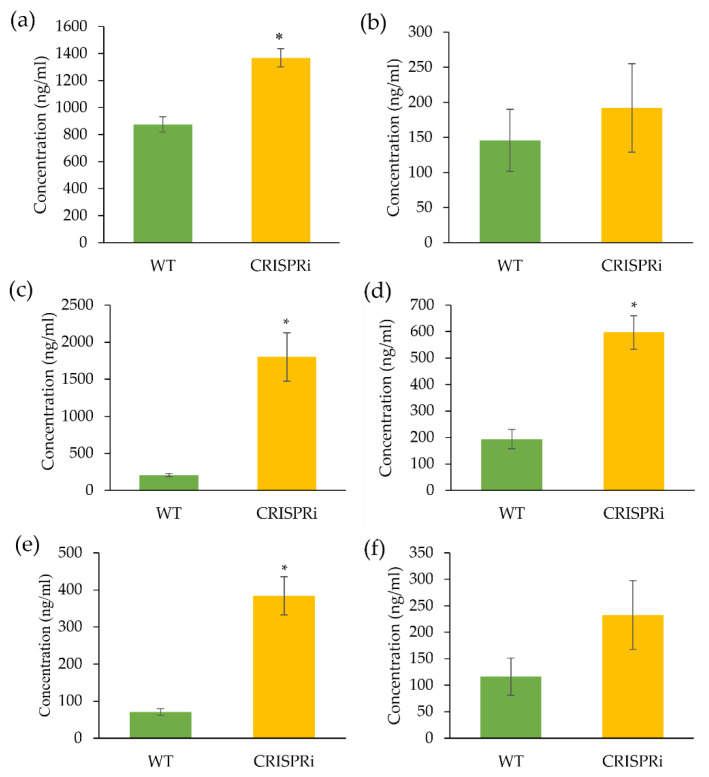
Concentration of selected compounds in wild type (WT) and transgenic CRISPRi cell lines of *Nicotiana tabacum* cell suspension cultures. (**a**) Cinnamic acid, (**b**) *p*-coumaric acid, (**c**) chlorogenic acid, (**d**) naringenin, (**e**) pinostrobin, and (**f**) kaempferol. Data represent the means of three biological replicates (n = 3). Error bars are standard deviation (±SD). Asterisk (*) represents significant differences at *p* < 0.05 according to paired *t*-test.

The accumulation of several flavonoids in wild-type and *C4H-*silenced cells was analyzed using LC-MS. As expected, the accumulation of cinnamic acid in the *C4H-*silenced cells was significantly higher (*p* < 0.05) than in the wild-type (Figure 5a). However, there was no significant difference in the product of the C4H enzyme, i.e., *p*-coumaric acid, between wild-type and *C4H-*silenced cells (Figure 5b). The downstream flavonoid products, namely, pinostrobin, naringenin, chlorogenic acid, and kaempferol, have also been determined. We found that the *C4H-*silenced cells produced a significantly higher (*p* < 0.05) concentration of chlorogenic acid, pinostrobin, and naringenin compared to the wild-type, with a total amount of 1799.69 ng/mL, 384.19 ng/mL, and 597.53 ng/mL, respectively (Figure 5c–f). The kaempferol was an exception, where no significant difference between the wild-type and the *C4H-*silenced cells was observed. 

## 4. Discussion

### 4.1. The CRISPRi System Is a Powerful Tool for Silencing C4H

Metabolic engineering approaches have been demonstrated as an effective way of increasing the production of flavonoids [35]. While the application of CRISPR/Cas9 technology for manipulating the metabolite biosynthetic pathways in microorganism cells, such as bacteria and fungi, has become a widely used technique [36,37,38], the use of this technology for the same purpose in plants is scarce. In this study, we demonstrated how a CRISPRi silencing approach could be used to silence our target gene, i.e., *NtC4H*, and determine if the silenced gene could affect flavonoid production. To achieve this goal, we first constructed the CRISPRi vector and introduced it into *N. tabacum* cell suspension cultures. We used cell suspension cultures since they are easy for transformation and harvesting, and suitable for studying complex processes at the molecular and cellular levels in controlled conditions [39]. 

Our results show that the *C4H-*silenced cells had a lower expression of *NtC4H* than the wild-type, suggesting that the CRISPRi approach effectively reduces the *NtC4H* transcript. The low *C4H* expression in the transformed cell lines also indicates that the designed sgRNAs efficiently targeted the promoter of the *NtC4H*, and three sgRNAs could increase the gene silencing efficiency. Similar findings have also been reported by Lowder et al. [40], where the author found that their dCas9-based transcriptional system, together with three sgRNA cassettes, could increase the *AtPAP1* transcript by 2- to 7-fold in the transgenic *Arabidopsis*. CRISPRi can be used to repress the transcription of endogenous genes in plants [27], and its effectiveness has been demonstrated in Arabidopsis, rice, and tobacco. For example, Lowder et al. [40] employed a PCO-dCas9-3X synthetic transcriptional repressor to suppress the *AtCSTF64* gene in Arabidopsis. The authors showed that this dCas9 construct could decrease almost 60% of the *AtCSTF64* transcript level. In the past, RNA interference (RNAi) that uses small RNA molecules to inhibit the translation of the target proteins was commonly used to manipulate plant metabolite biosynthesis. For instance, Sykes et al. [41] attempted to silence the expression of *C4H* in the hybrid eucalyptus (*Eucalyptus urophylla × E. grandis*) using RNAi. The authors found that the *C4H*-silenced cells showed a 0.22-fold lower *C4H* transcript level than the control. Similarly, Kumar et al. [42] reported that the antisense technique decreased the *C4H* expression in *N. tabacum* by a 0.23-fold reduction in the transgenic lines. However, despite its efficiency in regulating gene expression, RNAi is hindered by its inconsistency, incompleteness of knockdowns, and the potential for non-specificity in gene targeting [43]. In comparison, our findings show that the CRISPRi approach is more straightforward and efficient in knocking down *NtC4H* than conventional gene-knockdown strategies. 

### 4.2. The Flavonoid-Related Genes Were Upregulated in the C4H-Silenced Cells

We found that the lower expression of the *NtC4H* increased *Nt4CL*, *NtCHS*, and *NtCHI* transcripts, although the enhancement was not significant for *NtCHI*. C4H is an important enzyme in converting cinnamic acid to *p*-coumaric acid at the second step in the phenylpropanoid pathway. Silencing this gene increased the competing enzyme transcript of *Nt4CL*, located at another branch point in the phenylpropanoid pathway, next to *NtC4H*. The 4CL enzyme exists in different isoforms and is produced depending on the needs of the plants [44]. This enzyme is essential for flavonoid and monolignol biosynthesis in plants [45]. For example, Yang et al. [46] show that overexpressing *4CL* in *Rehmannia glutinosa* significantly increased flavonoid production. Similarly, the correlation between enhanced *4CL* expression and flavonoid biosynthesis has been reported in rice [47], cottonwood [48], barley [49], and soybean [50]. 

The CHS enzyme catalyzes *p*-coumaroyl-CoA (a product from 4CL) and three malonyl-coenzyme A (CoA) thioesters to form the specific chalcones [51]. Overexpressing this gene in soybean increased flavonoid production, such as liquiritigenin, isoliquiritigenin, and isoliquiritin [52]. However, the expression of *NtCHI* was not influenced by silencing *NtC4H*, probably due to the distance of *CHI* from the *C4H* in the pathway. CHI catalyzes the formation of flavanone through an intramolecular cyclization reaction, converting the bicyclic chalcone into tricyclic (2S)-flavanone [52]. It is noteworthy that our results should be interpreted with caution because most enzymes are transcriptionally regulated in a coordinated manner. Furthermore, the regulatory mechanisms controlling their expression are complex and have yet to be fully understood. 

### 4.3. The Flavonoid Production Was Altered in the C4H-Silenced Cells

Enhancement of *trans*-cinnamic acid in the *C4H-*silenced cells may be correlated with the knockdown of *NtC4H*. This observation was in agreement with the previous study by Kumar et al. [53], where the authors showed that downregulating the *C4H* expression had increased the accumulation of cinnamic acid in *Artemisia annua*. In our case, the silencing of the competing enzyme, *NtC4H*, may have diverted most of the intermediates and boosted metabolic flux to the 4CL route. As shown in our metabolic analysis, the *C4H*-silenced cells produced a higher accumulation of pinostrobin (via the *4CL* route) than naringenin (via the *C4H* route). This finding was supported by our qPCR analysis, where the expression of *Nt4CL* was upregulated. Similar findings were reported by Gifford et al. [54], where more pinocembrin was detected (via the *4CL* route) after silencing the *C4H* in *Datisca glomerata*. However, despite reducing *NtC4H* expression, the accumulation of naringenin in the *C4H*-silenced cells was higher than in the wild-type. This might be due to the hydroxylation of CoA esters of cinnamoyl-CoA to coumaroyl-CoA by cinnamoyl-CoA 4-hydroxylase [55]. Furthermore, Liu et al. [56] reported that pinocembrin could be converted to naringenin through hydroxylation without altering pinocembrin-related gene expression. 

**Figure 6:**
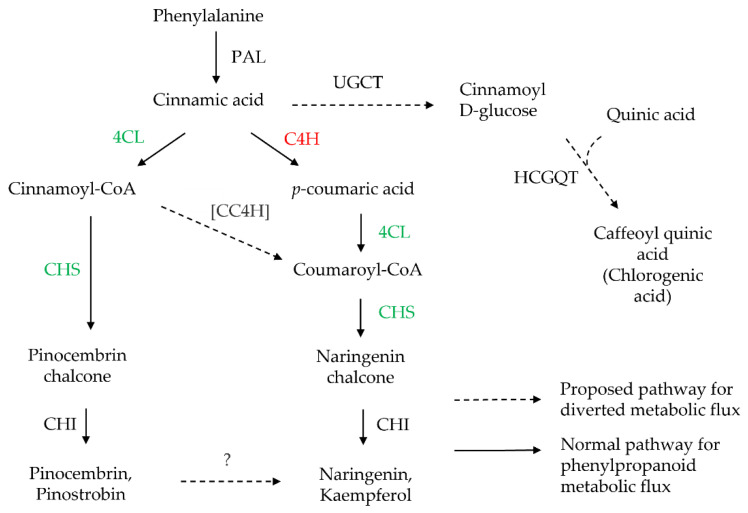
The proposed biosynthesis pathway of flavonoid in the *C4H*-silenced cells of *Nicotiana tabacum.* The dotted arrows show the potential diversion of metabolic flux. Alphabet in red indicates reduced expression, whereas alphabet in green indicates enhanced gene expression. PAL, phenylalanine ligase; C4H, cinnamate-4-hydroxylase; 4CL, 4-coumarate ligase; CHS, chalcone synthase; CHI, chalcone isomerase; UGCT, UDP-glucose:cinnamate glucosyltransferase; HCGQT, hydroxycinnamoyl glucose:quinate hydroxycinnamoyl transferase; CC4H, cinnamoyl-CoA 4-hydroxylase. The enzymes for 4-hydroxylation of cinnamoyl-CoA have not been demonstrated yet. Hence, CC4H is indicated in brackets showing their potential role.

Interestingly, chlorogenic acid increased (about 6-fold) when *NtC4H* expression was reduced, suggesting a possible metabolic flux diversion to the UDP-glucose:cinnamate glucosyltransferase (UGCT) pathway. UGCT catalyzes cinnamic acid to form cinnamoyl D-glucose and ultimately chlorogenic acid [57] (Figure 6). Chlorogenic acid is a major phenolic compound in plant metabolism as they are the precursor for lignin biosynthesis [58]. Besides, they are also actively involved in the plant’s defense mechanism against herbivores or pathogens [59] and protection against UV radiation [60]. Transcriptomic analysis by Xu et al. [61] revealed that *4CL* and *C4H* are highly associated with enhanced chlorogenic acid accumulation in sweet potatoes, indicating the involvement of the early enzymes of phenylpropanoid metabolism in chlorogenic acid biosynthesis.

## 5. Conclusions

This study examined the effects of *NtC4H* silencing in *N. tabacum* cell suspension cultures using the CRISPRi system. The reduction of *NtC4H* expression in the transgenic cells has upregulated their *Nt4CL* and *NtCHS* expression, causing the accumulation of pinostrobin via an alternate route. Besides, the *NtC4H* silencing might also divert the metabolic flux to chlorogenic acid synthesis. Hence, further research should profile the metabolites due to *NtC4H* silencing and examine the metabolic flux diversion using stable isotope tracers, such as ^13^C- and ^15^N-labelled substrates. In conclusion, the CRISPRi system could be used to silence *NtC4H*, and suppressing this gene could alter the flavonoid-related gene expression and production in *N. tabacum.*

## Figures and Tables

**Table 1:** List of plasmids for *Escherichia coli* transformation.

Plasmid	Backbone	Bacterial Antibiotic Resistance	Concentration of Antibiotics (mg/L)	Remarks
gRep1-TN	pYPQ131c	Tetracycline	5	1st gRNA expression vector for tobacco
gRep2-TN	pYPQ132c	Tetracycline	5	2nd gRNA expression vector for tobacco
gRep3-TN	pYPQ133c	Tetracycline	5	3rd gRNA expression vector for tobacco
pGGA3-TN	pYPQ143	Spectinomycin	100	gRNA entry vector for tobacco
pdCas9	pYPQ153	Spectinomycin	100	dCas9 entry vector
pCRi-0	pMDC32-Ubi1	Kanamycin	50	Plant destination vector/ CRISPRi empty vector
pCTN	pMDC32-Ubi1	Kanamycin	50	CRISPRi vector

**Table 2:** gRNA sequences cloned into their respective expression vector.

gRNA	gRNA Sequence (5′-3′)	Expression Vector	Plasmid
G1F-TN	GTG TGC GTT AAT ATT AAC GGA GAG TTG G	pYPQ131c	gRep1-TN
G1R-TN	AAA CCC AAC TCT CCG TTA ATA TTA ACG C		
G2F-TN	GTG TGC CTC ACA CTT TCT TAT CTT ATG G	pYPQ132c	gRep2-TN
G2R-TN	AAA CCC ATA AGA TAA GAA AGT GTG AGG C		
G3F-TN	GTG TGG AGA AAA GAA ACT TGG GAG TTG G	pYPQ133c	gRep3-TN
G3R-TN	AAA CCC AAC TCC CAA GTT TCT TTT CTC C		

**Table 3:** qPCR primer pairs for flavonoid-related genes.

Gene	Primer Name	Primer Sequence (5′-3′)
EF1α	K-EF1aF K-Efa1R	CCCTTGGTGTCAAGCAAATG GGTAGGAAGAAACCTCCTTCAC
LF25	K-L25F K-L25R	AAAGCTGATCCGTCCAAAAA GACAGCCTTGGCAACCTTAG
C4H	K-C4H F K-C4H R	GGAAGAAGCCCGAAGAGTTTAG CTCCTCCTACCAACACCAAATG
4CL	4CL-F 4CL-R	GGTTACACACTGGCGACATTGG GGAACTTCTCCTGCTTGCTCATC
CHS	K-CHS_TN F K-CHS_TN R	CCTTTGTTCGAGCTTGTCTCTG GCCCAGGAACATCTTTGAGTAAG
CHI	K-CHI_TN F K-CHI_TN R	ATCCAGTGATTGAGGAGAAACC TCAGGCTCAGTTGACAAAGG

## Data Availability

The data in this study are available upon request from the corresponding author.

## Supplementary Materials

The following supporting information can be downloaded at: https://www.mdpi.com/article/10.3390/biology11081127/s1, Figure S1: Sequencing of sgRNA sequence in (a) gRNA expression vectors (gRep1-TN, gRep2-TN, and gRep3-TN) and (b) gRNA entry vectors (pGGA3-TN). Sequence highlighted indicates the sgRNA sequences; Figure S2: The 405 bp sequence from the sequencing of CRISPRi vector, pCTN. Sequence from dCas9 plasmid highlighted in orange; Sequences of gRNA highlighted in blue; Sequences from plant expression vector highlighted in green.
