# Supplementary material for: CRISPRi-Mediated Down-Regulation of the Cinnamate-4-Hydroxylase (C4H) Gene Enhances the Flavonoid Biosynthesis in Nicotiana tabacum
Source: Biology (Basel). 2022 Jul 27;11(8):1127. doi: 10.3390/biology11081127 (PMC9404795; doi:10.3390/biology11081127)
Supplement: Supplementary file 1 [file biology-11-01127-s001.zip › Supplementary material.pptx]

## Slide 1
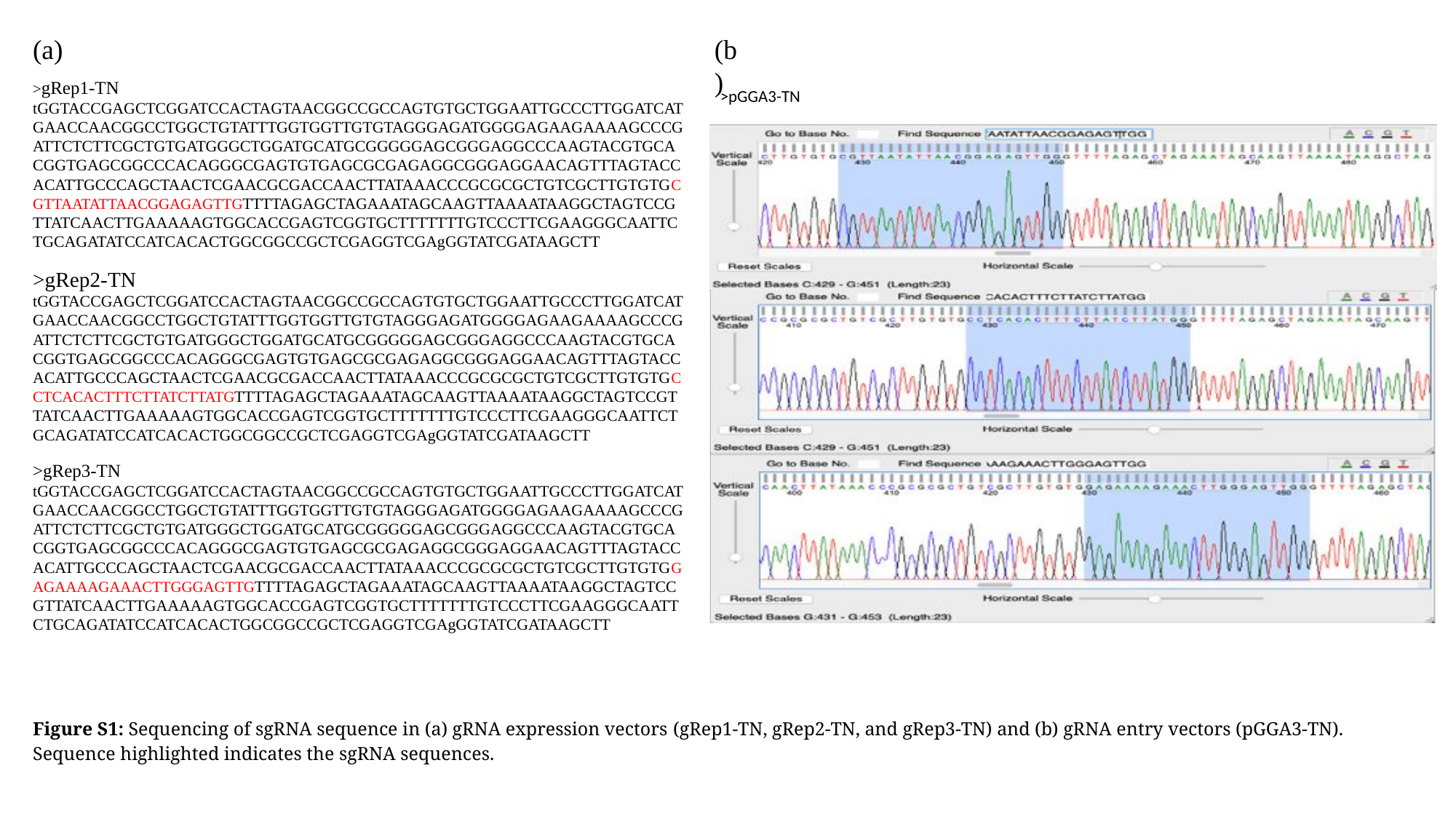

(a)
(b)
>gRep1-TN
tGGTACCGAGCTCGGATCCACTAGTAACGGCCGCCAGTGTGCTGGAATTGCCCTTGGATCATGAACCAACGGCCTGGCTGTATTTGGTGGTTGTGTAGGGAGATGGGGAGAAGAAAAGCCCGATTCTCTTCGCTGTGATGGGCTGGATGCATGCGGGGGAGCGGGAGGCCCAAGTACGTGCACGGTGAGCGGCCCACAGGGCGAGTGTGAGCGCGAGAGGCGGGAGGAACAGTTTAGTACCACATTGCCCAGCTAACTCGAACGCGACCAACTTATAAACCCGCGCGCTGTCGCTTGTGTGCGTTAATATTAACGGAGAGTTGTTTTAGAGCTAGAAATAGCAAGTTAAAATAAGGCTAGTCCGTTATCAACTTGAAAAAGTGGCACCGAGTCGGTGCTTTTTTTGTCCCTTCGAAGGGCAATTCTGCAGATATCCATCACACTGGCGGCCGCTCGAGGTCGAgGGTATCGATAAGCTT
>gRep2-TN
tGGTACCGAGCTCGGATCCACTAGTAACGGCCGCCAGTGTGCTGGAATTGCCCTTGGATCATGAACCAACGGCCTGGCTGTATTTGGTGGTTGTGTAGGGAGATGGGGAGAAGAAAAGCCCGATTCTCTTCGCTGTGATGGGCTGGATGCATGCGGGGGAGCGGGAGGCCCAAGTACGTGCACGGTGAGCGGCCCACAGGGCGAGTGTGAGCGCGAGAGGCGGGAGGAACAGTTTAGTACCACATTGCCCAGCTAACTCGAACGCGACCAACTTATAAACCCGCGCGCTGTCGCTTGTGTGCCTCACACTTTCTTATCTTATGTTTTAGAGCTAGAAATAGCAAGTTAAAATAAGGCTAGTCCGTTATCAACTTGAAAAAGTGGCACCGAGTCGGTGCTTTTTTTGTCCCTTCGAAGGGCAATTCTGCAGATATCCATCACACTGGCGGCCGCTCGAGGTCGAgGGTATCGATAAGCTT
>gRep3-TN
tGGTACCGAGCTCGGATCCACTAGTAACGGCCGCCAGTGTGCTGGAATTGCCCTTGGATCATGAACCAACGGCCTGGCTGTATTTGGTGGTTGTGTAGGGAGATGGGGAGAAGAAAAGCCCGATTCTCTTCGCTGTGATGGGCTGGATGCATGCGGGGGAGCGGGAGGCCCAAGTACGTGCACGGTGAGCGGCCCACAGGGCGAGTGTGAGCGCGAGAGGCGGGAGGAACAGTTTAGTACCACATTGCCCAGCTAACTCGAACGCGACCAACTTATAAACCCGCGCGCTGTCGCTTGTGTGGAGAAAAGAAACTTGGGAGTTGTTTTAGAGCTAGAAATAGCAAGTTAAAATAAGGCTAGTCCGTTATCAACTTGAAAAAGTGGCACCGAGTCGGTGCTTTTTTTGTCCCTTCGAAGGGCAATTCTGCAGATATCCATCACACTGGCGGCCGCTCGAGGTCGAgGGTATCGATAAGCTT
>pGGA3-TN
Figure S1: Sequencing of sgRNA sequence in (a) gRNA expression vectors (gRep1-TN, gRep2-TN, and gRep3-TN) and (b) gRNA entry vectors (pGGA3-TN). Sequence highlighted indicates the sgRNA sequences.

## Slide 2
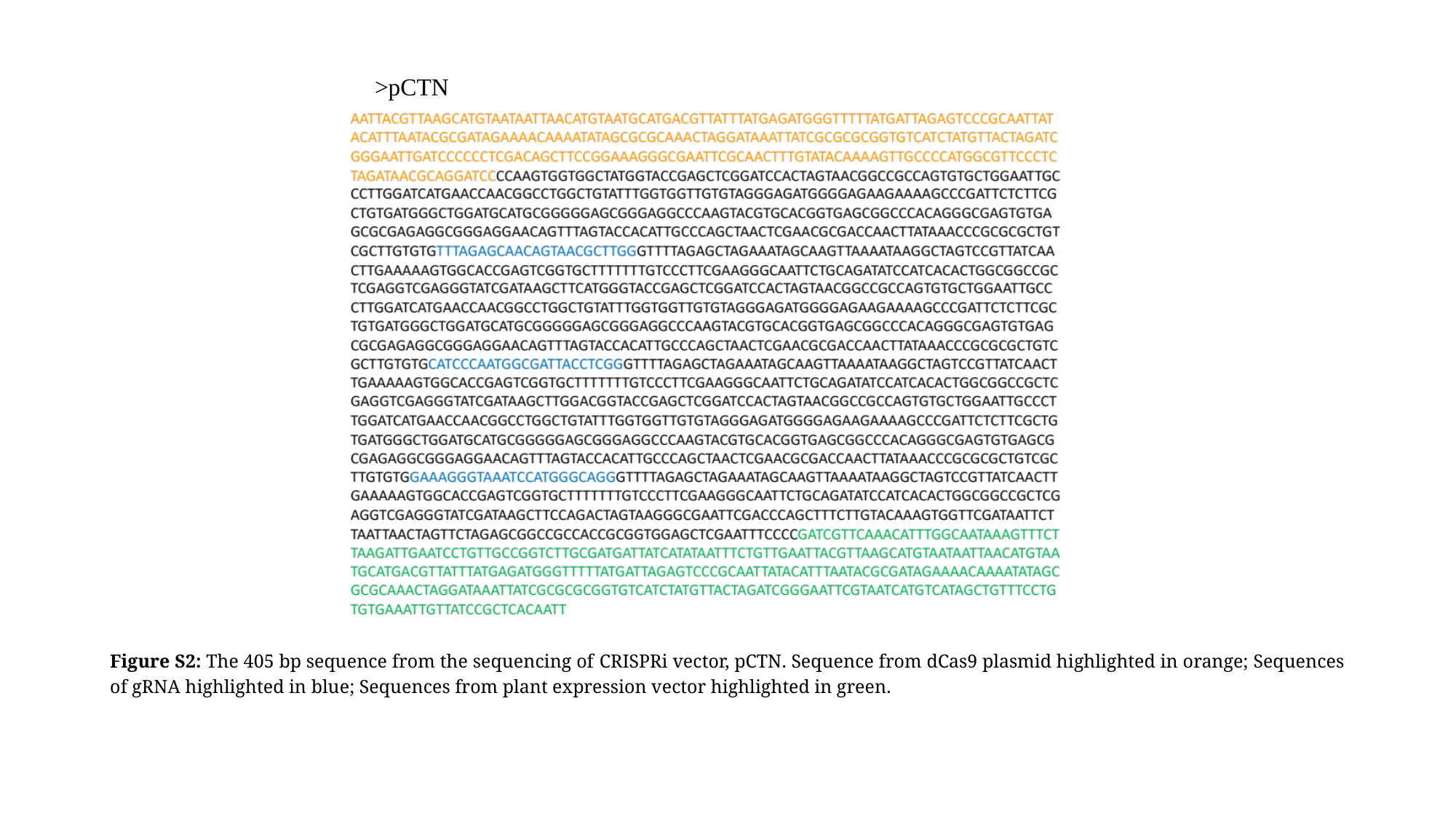

>pCTN
Figure S2: The 405 bp sequence from the sequencing of CRISPRi vector, pCTN. Sequence from dCas9 plasmid highlighted in orange; Sequences of gRNA highlighted in blue; Sequences from plant expression vector highlighted in green.
